# Supplementary material for: A tessellation-based colocalization analysis approach for single-molecule localization microscopy
Source: Nat Commun. 2019 May 30;10:2379. doi: 10.1038/s41467-019-10007-4 (PMC6542817; doi:10.1038/s41467-019-10007-4)
Supplement: Supplementary file 1 — Supplementary Information [file 41467_2019_10007_MOESM1_ESM.pdf]

Supplementary Information

A tessellation-based colocalization analysis for single-molecule localization microscopy

Levet et al.

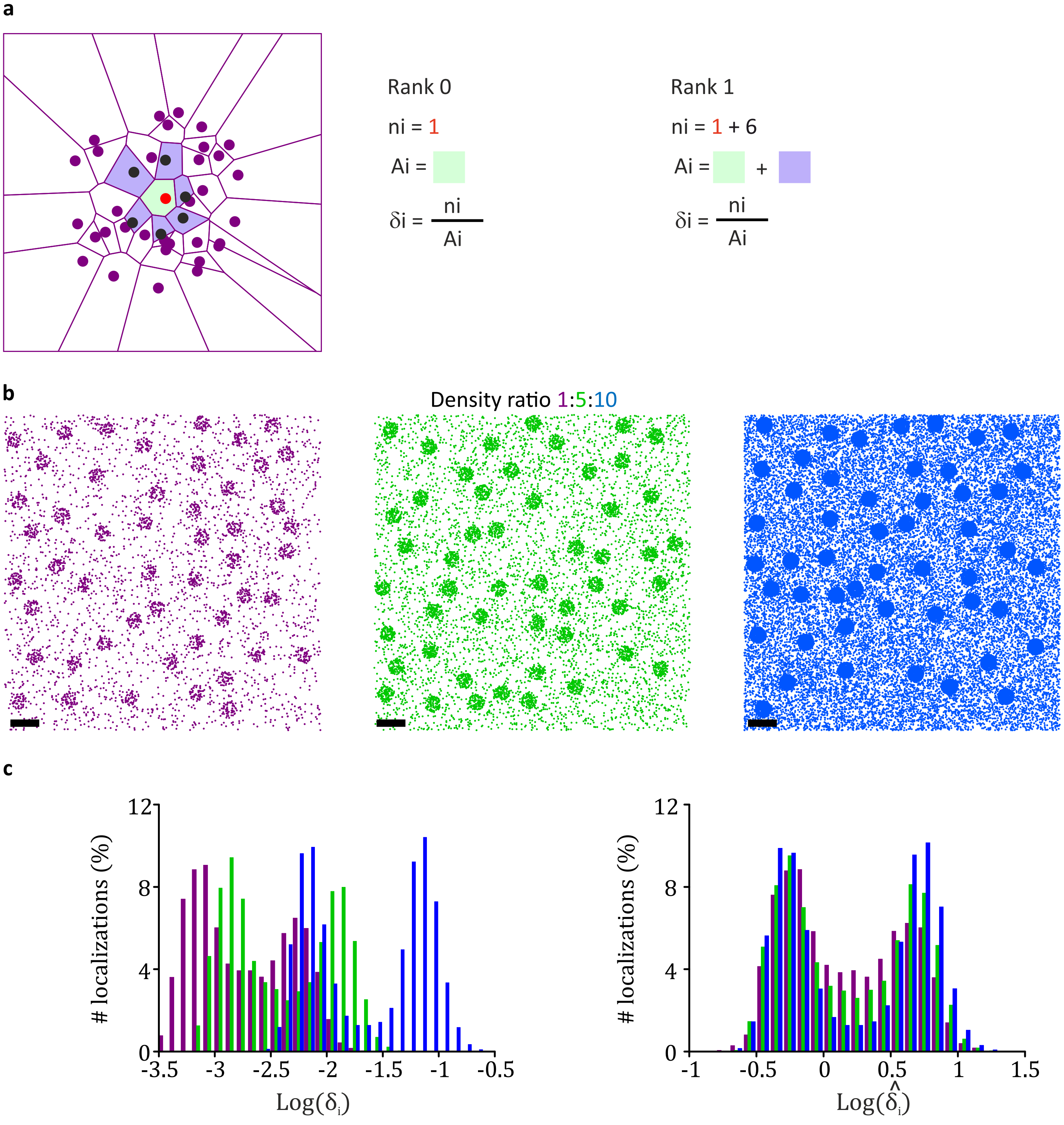

Supplementary Figure 1: Voronoï-based computation and normalization of the density. (a) The 1st rank density δ_i of a localization i (red) is defined as the number of direct neighbors (black) plus one divided by the total area of all the corresponding polytopes (green and purple). (b) 3 simulations with an enrichment factor R = 10 and 3 different density ratios 1:1, 1:5 and 1:10 corresponding to cluster densities of 0.0065 mol.nm^{-2} (left), 0.013 mol.nm^{-2} (middle) and 0.065 mol.nm^{-2} (right); scale bar = 200 nm. (c) Distribution of the localization densities before (left) and after normalization (right). After normalization, the distributions of the 3 simulations are perfectly aligned.

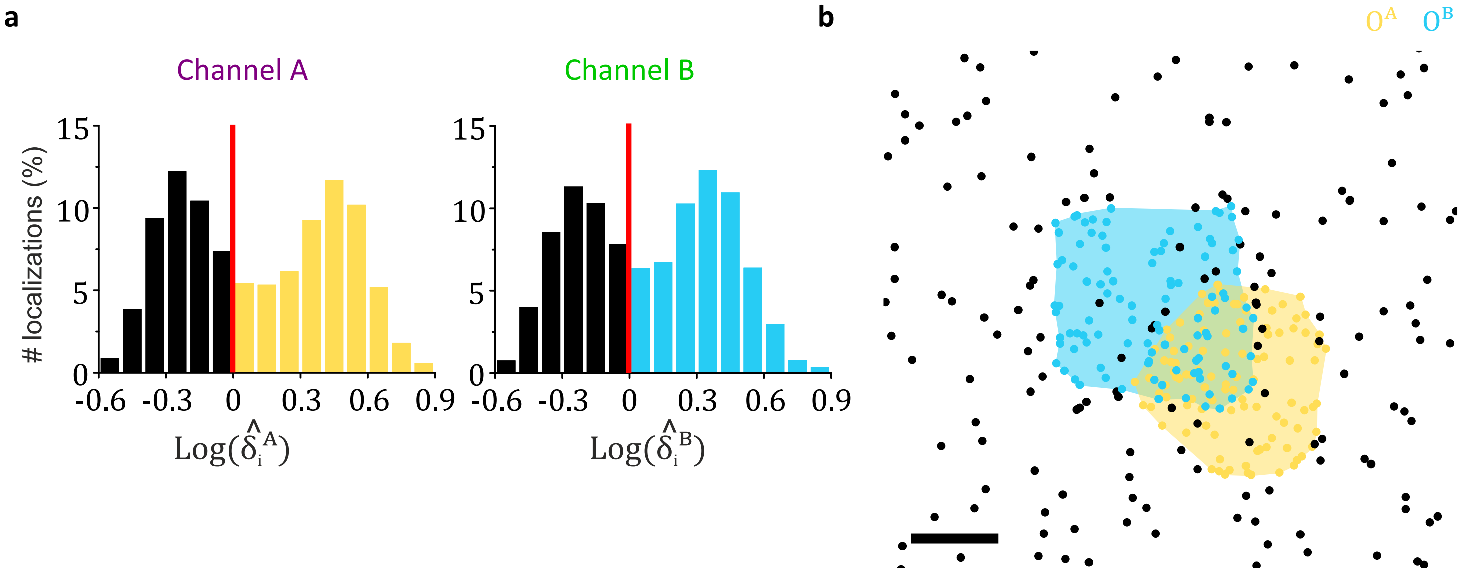

Supplementary Figure 2: Object-based colocalization analysis. (a) Automatic thresholding using $\hat{\delta}_i>1$ and 3-class classification of the localizations of the two channels. High density localizations in channel A (resp. B) are colored in orange (resp. cyan) while background localizations are colored in black. (b) Segmentation and computation of the colocalization as the overlapping surface between the segmented objects of the 2 channels (scale bar 50 nm).

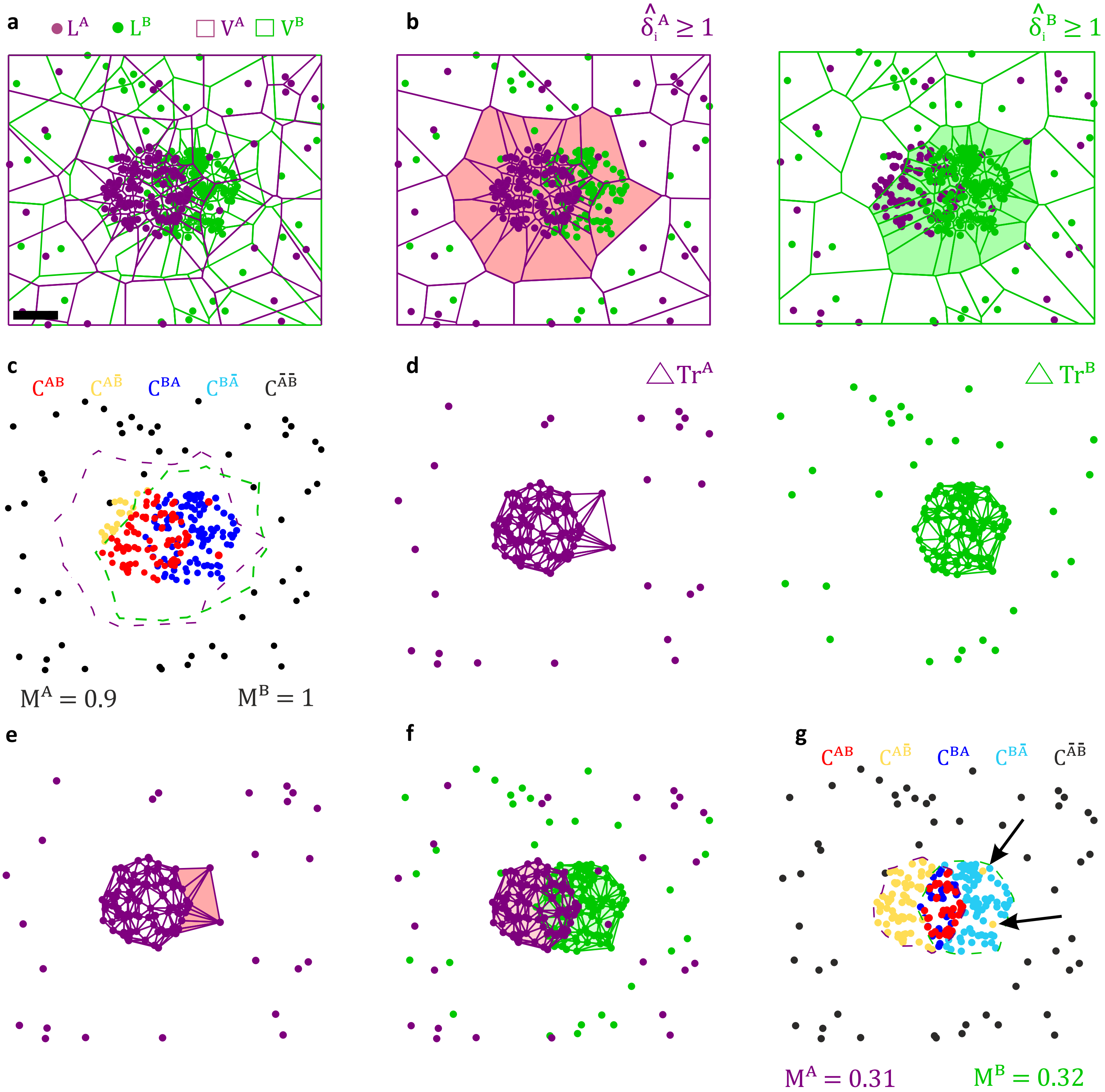

Supplementary Figure 3: Voronoï edge correction. (a) Simulation of two partially colocalized 100 nm clusters with an inter-distance of 50 nm together with their corresponding Voronoï diagrams (scale bar 50 nm). (b) Segmentation of the clustered localizations of the 2 channels with $\hat{\delta}_i$ >1. (c) Five class classification of the localizations without edge correction. The magenta and green dashed lines represent the large influence region of the clustered localizations' polygons, overestimating the colocalization values (M^A = 0.9 and M^B = 1). (d) Triangle sets Tr^A and Tr^B describing the two high-density classes C^A and C^B. (e) Outliers (pink triangles) removal from Tr^A. (f) Corrected triangle sets Tr^A and Tr^B. (g) Pointed localizations have been transferred from C^{AB} to $C^{A\bar{B}}$, resulting in correcting both the influence region (dashed lines) and the colocalization values (M^A = 0.31 and M^B = 0.32).

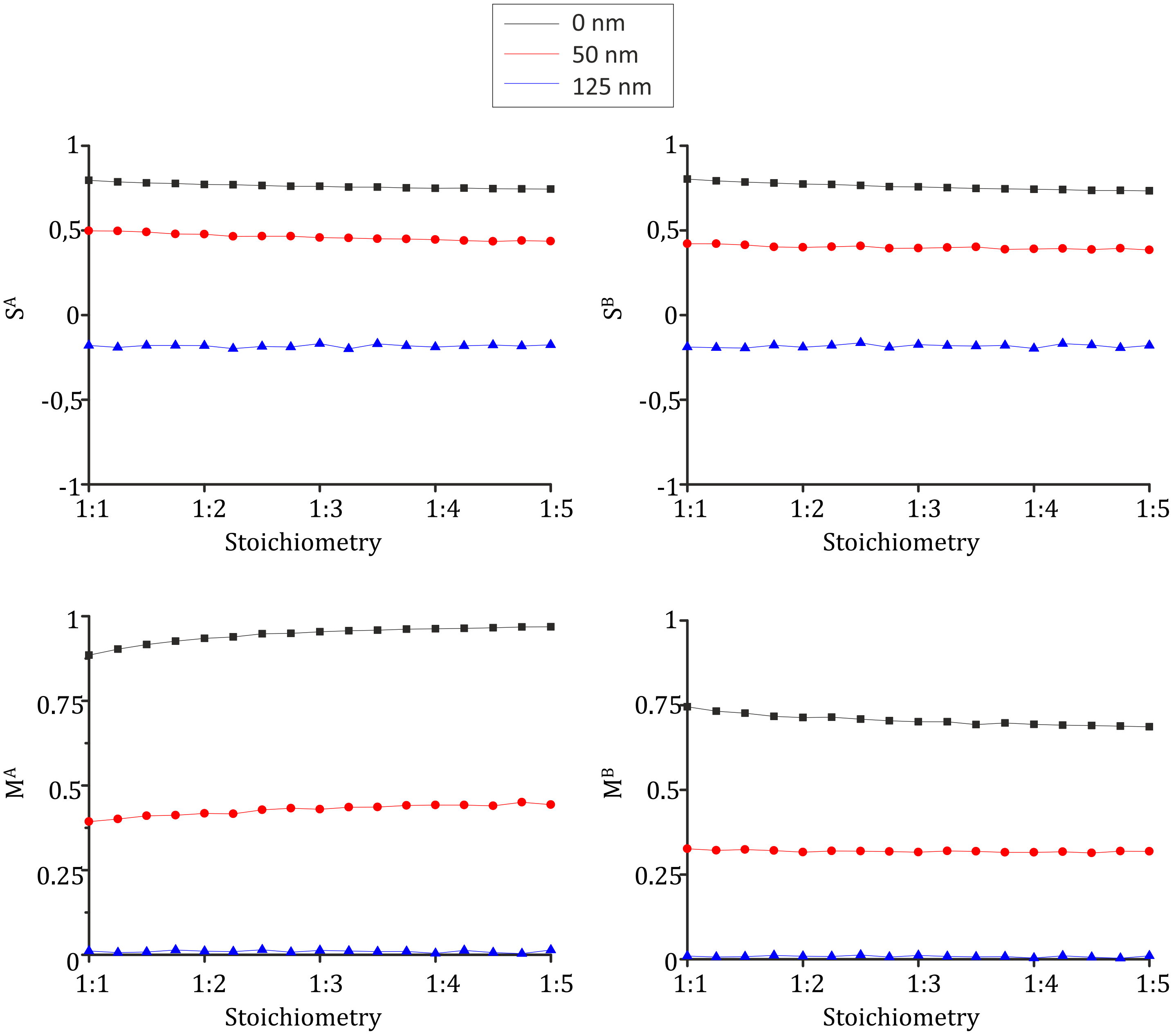

Supplementary Figure 4: Robustness of Coloc-Tesseler with respect to the relative density. Simulations of 100 randomly placed, non-overlapping clusters of 100 nm circular (channel A) and square (channel B) clusters. Channel A has a fixed cluster density 0.013 mol.nm^{-2}, while channel B has a varying cluster density ranging from 0.013 mol.nm^{-2} to 0.065 mol.nm^{-2} with 0.0026 mol.nm^{-2} steps, corresponding to ratios ranging from 1:1 to 5:1 with 20 steps. The robustness of Color-Tesseler with respect to the relative density is illustrated by the very small SEM of the quantifications (d = 0 nm: M^A = 0.94 ± 0.006 SEM, M^B = 0.7 ± 0.004 SEM, S^A = 0.76 ± 0.003 SEM, S^B = 0.76 ± 0.005 SEM; d = 50 nm: M^A = 0.43 ± 0.004 SEM, M^B = 0.32 ± 0.001 SEM, S^A = 0.46 ± 0.005 SEM, S^B = 0.4 ± 0.003 SEM; d = 125 nm: M^A = 0.01 ± 0.001 SEM, M^B = 0.01 ± 0.0001 SEM, S^A = -0.18 ± 0.002 SEM, S^B = -0.18 ± 0.002 SEM).

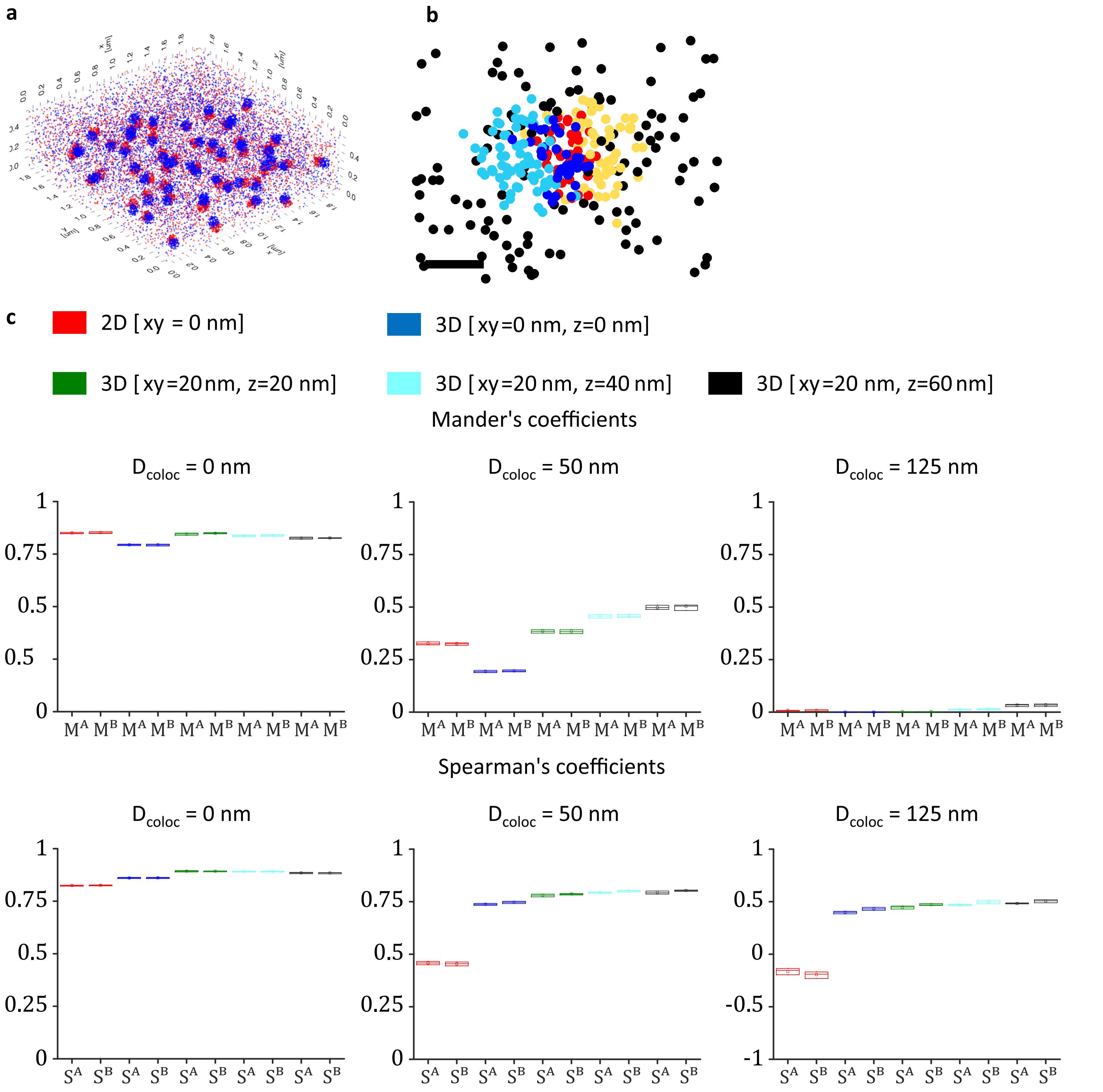

Supplementary Figure 5: Colocalization analysis of 3D simulation data. (a) Visualization, using VISP software[1], of a two-color 3D simulation composed of 100 nm diameter spherical clusters with inter-distance d=50 nm. (b) Two-dimensional projection of the five class classification molecules using Coloc-Tesseler (scale bar 50 nm). (c) Colocalization analysis of 2D and 3D simulation data with various localization accuracies C1=(Δ_{xy} = 20 nm, Δ_z = 20 nm), C2=(Δ_{xy} = 20 nm, Δ_z = 40 nm) and C3=(Δ_{xy} = 20 nm, Δ_z = 60 nm) and 3 different clusters' inter-distances d of 0 nm, 50 nm and 125 nm. Compared to 2D, 3D anisotropic localization precision just barely degrades the colocalization values, with a maximum of 6% difference for d=0 (fully overlapping clusters), 17% for d=50nm (partially overlapping), and 2.5% for d=125nm (non-overlapping). The fluctuation amplitude is computed as $|M_{2D} – M_{3D}|$*100. In all box plots the center line is the median, the square is the mean and the bounds of the boxes are the 75 and 25% percentiles i.e., the interquartile range (IQR).

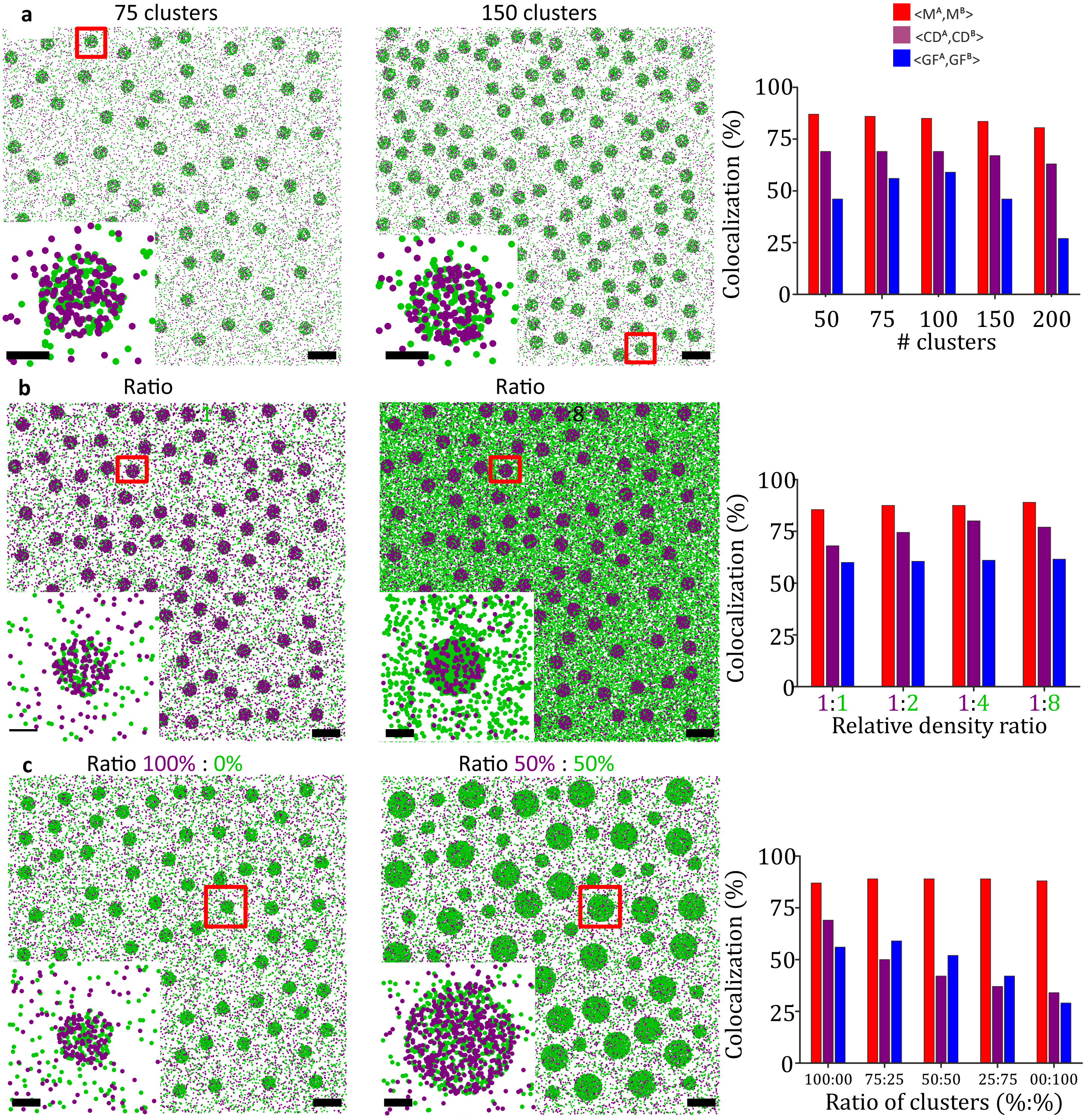

Supplementary Figure 6: Comparison of Coloc-Tesseler with Clus-DoC[2] and Getis & Franklin[3]. (a) Simulations of randomly placed fully colocalized clusters, with varying number of 100 nm diameter clusters (50, 75, 100, 150 and 200). Examples with 75 (left) and 150 (middle) clusters. The colocalization values obtained with the 3 techniques (right) illustrate the robustness of Coloc-Tesseler (CT) and Clus-DoC (CD) but the sensitivity of Getis & Franklin (GF) to the density of clusters. (b) Simulations of 100 randomly placed fully colocalized 100 nm clusters, with varying relative density ratios (1:1, 1:2, 1:4 and 1:8). Examples with density ratios of 1:1 (left) and 1:8 (middle). The colocalization computed with the three techniques (right) show that both CT and GF display high robustness, while CD exhibits a higher variability. Despite the higher variability of CT compared to GF, CT exhibits the best colocalization value. (c) Simulations of 75 randomly placed fully colocalized clusters with varying percentage of 100 nm and 200 nm diameter clusters (100:0, 75:25, 50:50, 25:75, 0:100). Examples with cluster ratios of 100:0 (left) and 50:50 (middle). Colocalization computed with the three techniques (right) illustrates again the robustness of Coloc-Tesseler with a strong stability and a high colocalization value. On the contrary, both GF and CD display a high sensitivity to this parameter with low colocalization values. Scale bars for the entire datasets = 200 nm, scale bars for the magnification = 50 nm.

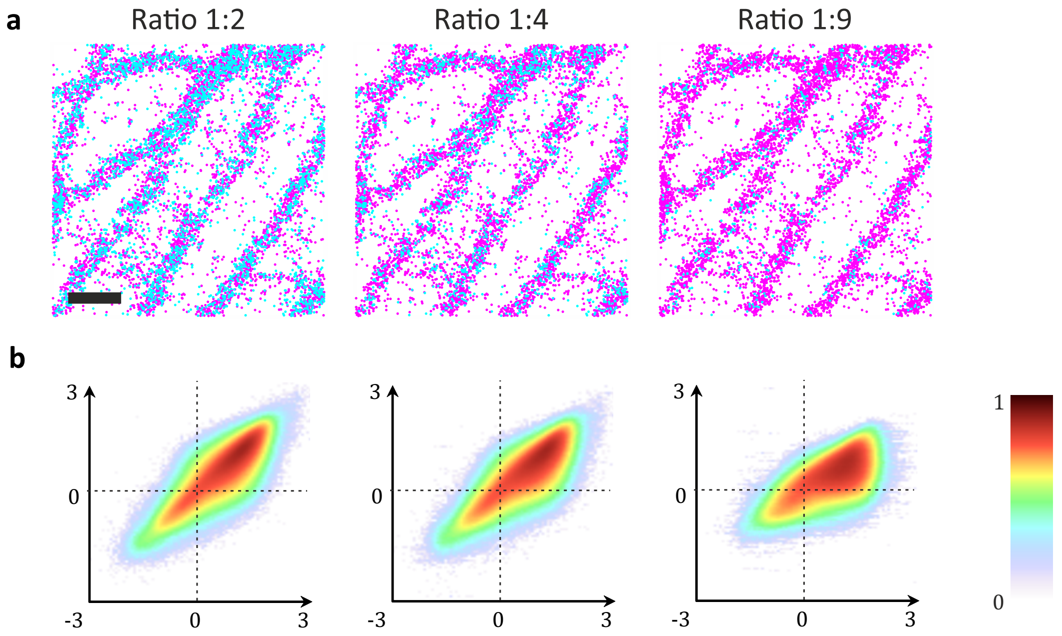

Supplementary Figure 7: Experimental datasets with varying densities. (a) Two-colors point rendering of the same microtubule dataset with varying relative densities (left 33%:67%, middle 20%:80%, right 10%:90%) (scale bar 250 nm). (b) Corresponding scatterplots of the normalized pair-densities.

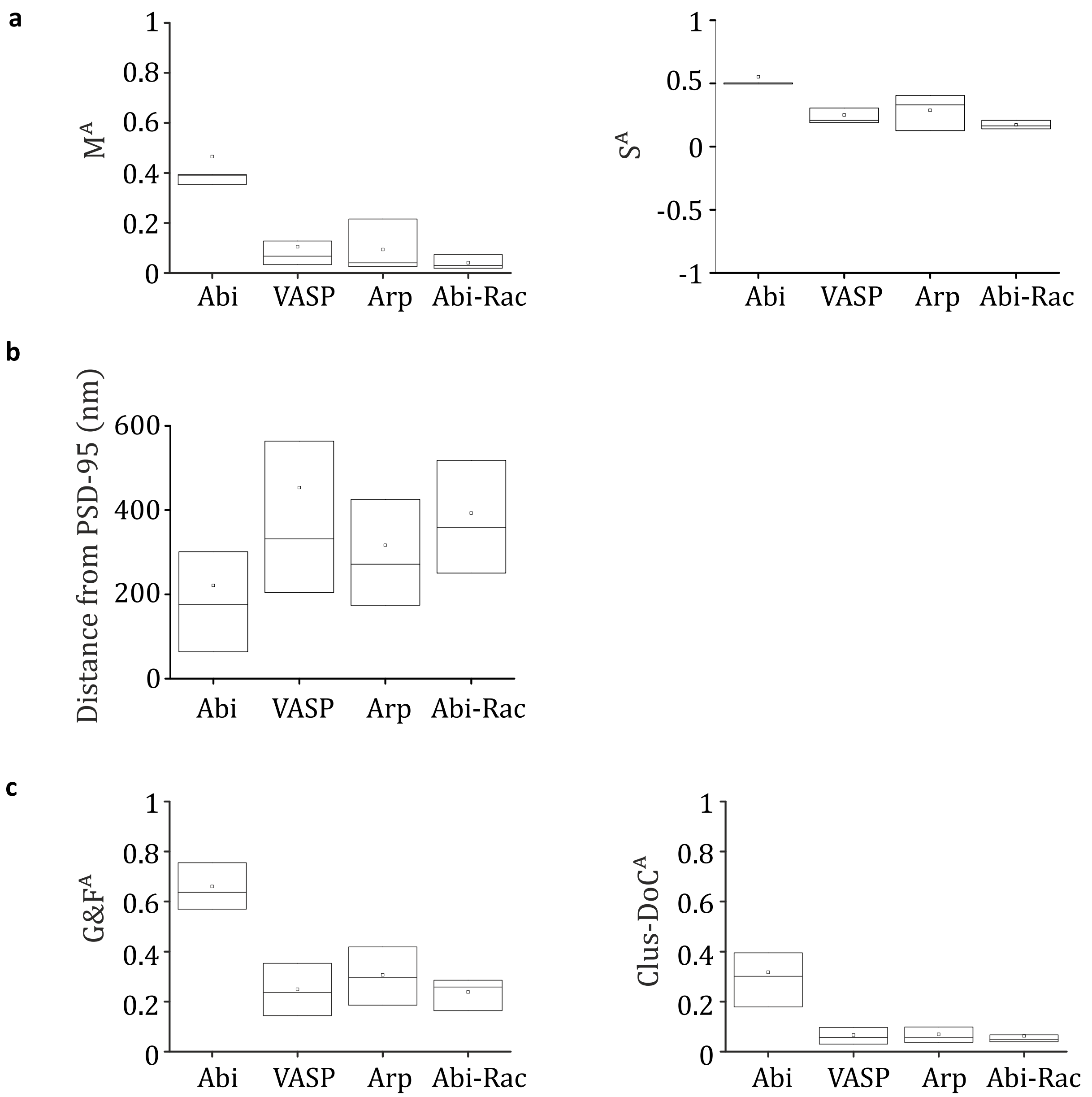

Supplementary Figure 8: Colocalization analysis of F-actin regulators and PSD95. (a) Manders' coefficient computed on the whole datasets for the different F-actin regulatory proteins (Abi1: M^A = 0.546 ± 0.09 SEM; VASP: M^A = 0.1 ± 0.03 SEM; ArpC5A: M^A = 0.09 ± 0.06 SEM; Abi1 with constitutive Rac1 activation: M^A = 0.04 ± 0.02 SEM) (left). Spearman rank correlation computed on the same datasets (Abi1: S^A = 0.55 ± 0.05 SEM; VASP: S^A = 0.25 ± 0.03 SEM; ArpC5A: S^A = 0.28 ± 0.09 SEM; Abi1 with constitutive Rac1 activation: S^A = 0.17 ± 0.02 SEM) (right). (b) Distance between the barycenters of the F-actin and PSD95 clusters (Abi1: 221 ± 21 nm, VASP: 453 ± 19 nm, ArpC5A: 317 ± 18 nm, Abi1 with constitutive Rac1 activation: 393 ± 22 nm). (c) Analysis of the same datasets using Getis&Franklin (left, Abi1: GF^A = 66.06% ± 1.81 SEM; VASP: GF^A = 24.9% ± 1.55 SEM; ArpC5A: GF^A = 30.63% ± 2.96 SEM; Abi1 with constitutive Rac1 activation: GF^A = 23.84% ± 1.65 SEM) and ClusDoc (right, Abi1: CD^A = 31.71% ± 2.4 SEM; VASP: CD^A = 6.62% ± 0.55 SEM; ArpC5A: CD^A = 6.88% ± 0.78 SEM; Abi1 with constitutive Rac1 activation: CD^A = 6.26% ± 1.01 SEM) colocalization methods. In all box plots the center line is the median, the square is the mean and the bounds of the boxes are the 75 and 25% percentiles i.e., the interquartile range (IQR).

Supplementary References

1. Beheiry, M.E. & Dahan, M. ViSP: representing single-particle localizations in three dimensions. *Nat Meth* 10, 689-690 (2013).
2. Pageon, S.V., Nicovich, P.R., Mollazade, M., Tabarin, T., Gaus, K. Clus-DoC: A combined cluster detection and colocalization analysis for single-molecule localization microscopy data. *Mol. Biol. Cell.* 27(22), 3627-3636 (2016).
3. Rossy, J., Cohen, E., Gaus, K., Owen, DM. Method for co-cluster analysis in multichannel single-molecule localisation data. *Histochem Cell Biol.* 141, 605-12 (2014).
